# Mouse large-scale phenotyping initiatives: overview of the European Mouse Disease Clinic (EUMODIC) and of the Wellcome Trust Sanger Institute Mouse Genetics Project

**DOI:** 10.1007/s00335-012-9418-y

**Published:** 2012-09-09

**Authors:** Abdel Ayadi, Marie-Christine Birling, Joanna Bottomley, James Bussell, Helmut Fuchs, Martin Fray, Valérie Gailus-Durner, Simon Greenaway, Richard Houghton, Natasha Karp, Sophie Leblanc, Christoph Lengger, Holger Maier, Ann-Marie Mallon, Susan Marschall, David Melvin, Hugh Morgan, Guillaume Pavlovic, Ed Ryder, William C. Skarnes, Mohammed Selloum, Ramiro Ramirez-Solis, Tania Sorg, Lydia Teboul, Laurent Vasseur, Alison Walling, Tom Weaver, Sara Wells, Jacqui K. White, Allan Bradley, David J. Adams, Karen P. Steel, Martin Hrabě de Angelis, Steve D. Brown, Yann Herault

**Affiliations:** 1 Institut Clinique de la Souris, PHENOMIN, IGBMC/ICS-MCI, CNRS, INSERM, Université de Strasbourg, UMR7104, UMR964, 1 rue Laurent Fries, 67404 Illkirch, France; 2 The Wellcome Trust Sanger Institute, Wellcome Trust Genome Campus, Hinxton, Cambridge, CB10 1SA UK; 3 German Mouse Clinic, Institute of Experimental Genetics, Helmholtz Zentrum München, German Research Center for Environmental Health (GmbH), Ingolstädter Landstraße 1, 85764 Neuherberg/Munich, Germany; 4 Medical Research Council Harwell (Mammalian Genetics Unit and Mary Lyon Centre), Harwell, Oxfordshire OX11 0RD UK; 5 Chair of Experimental Genetics, Center of Life and Food Sciences Weihenstephan, TUM, 85350 Freising-Weihenstephan, Germany

## Abstract

Two large-scale phenotyping efforts, the European Mouse Disease Clinic (EUMODIC) and the Wellcome Trust Sanger Institute Mouse Genetics Project (SANGER-MGP), started during the late 2000s with the aim to deliver a comprehensive assessment of phenotypes or to screen for robust indicators of diseases in mouse mutants. They both took advantage of available mouse mutant lines but predominantly of the embryonic stem (ES) cells resources derived from the European Conditional Mouse Mutagenesis programme (EUCOMM) and the Knockout Mouse Project (KOMP) to produce and study 799 mouse models that were systematically analysed with a comprehensive set of physiological and behavioural paradigms. They captured more than 400 variables and an additional panel of metadata describing the conditions of the tests. All the data are now available through EuroPhenome database (www.europhenome.org) and the WTSI mouse portal (http://www.sanger.ac.uk/mouseportal/), and the corresponding mouse lines are available through the European Mouse Mutant Archive (EMMA), the International Knockout Mouse Consortium (IKMC), or the Knockout Mouse Project (KOMP) Repository. Overall conclusions from both studies converged, with at least one phenotype scored in at least 80 % of the mutant lines. In addition, 57 % of the lines were viable, 13 % subviable, 30 % embryonic lethal, and 7 % displayed fertility impairments. These efforts provide an important underpinning for a future global programme that will undertake the complete functional annotation of the mammalian genome in the mouse model.

## Introduction

Sequencing a genome will soon be routinely achieved in less than a week. Nevertheless, despite advances in our ability to decipher and annotate genomes, our understanding of the functions of genes is limited. With its highly developed genetics, the mouse has become the mammalian model of choice for the study of gene function (Collins et al. 2007). Almost one third of the known mouse genes representing predictive forms of a coding sequence are still not well annotated for functions. A third of mouse genes possess some functional annotation, reflecting the analysis of point mutations, though not always related to loss-of-function effects. The analysis of the remaining third has depended on the specific interests and expertise of investigators and not, in most cases, on a comprehensive phenotyping approach.

Several initiatives have combined effort over the last few years to attempt a systematic and comprehensive functional assessment of all mouse genes. Based on a roadmap discussed in 2004 (Austin et al. 2004; Auwerx et al. 2004), a series of mouse programmes have been set up with the goal of generating a compendium of mutations in embryonic stem (ES) cells, to produce the mutant mouse lines, to phenotype them, and to make these new resources available to the scientific community (Table 1). The first element of this vision, the construction of a mutant ES cell resource for every mouse gene, has led to the development of the International Knock-out Mouse Consortium (IKMC), which brings together the various programmes funded in Europe (European Conditional Mouse Mutagenesis, EUCOMM), the US (Knock-Out Mouse Project, KOMP, and Texas A&M Institute for Genomic Medicine, TIGM), Canada (North American Conditional Mouse Mutagenesis Project, NorCOMM), and Asia (The Asian Mouse Mutagenesis and Resource Association, AMMRA). This consortium began its work in 2006 and aims to generate a complete resource of reporter-targeted alleles in C57BL/6 N (B6 N) mouse ES cells. The C57BL/6 genetic background is considered to be the ideal background for large-scale phenotyping with a highly characterized genome and one of the best characterised inbred strains, although resistant to certain complex traits such as tumour formation. The design and construction of the conditional targeting constructs was performed through a well-defined pipeline (Skarnes et al. 2011).

**Table 1:** List of the consortia connected to the mouse large-scale phenotyping initiatives

Consortium	Website
The European Conditional Mouse Mutagenesis Programme (EUCOMM)	www.eucomm.org
The EUCOMMTools: Tools for Functional Annotation of the Mouse Genome (EUCOMMTools)	www.eucommtools.org
The European Mouse Phenotyping Resource of Standardised Screens (EMPRESS)	www.empress.har.mrc.ac.uk
The European Union Mouse Research for Public Health and Industrial Applications (EUMORPHIA)	www.eumorphia.org
The European Mouse Disease Clinic (EUMODIC)	www.EUMODIC.org
The EuroPhenome Mouse Phenotyping Resource	www.europhenome.org
The European Mouse Mutant Archive (EMMA)	www.emmanet.org
The European Infrastructure for Phenotyping and Archiving of model mammalian genomes (INFRAFRONTIER)	www.infrafrontier.eu
The International Knockout Mouse Consortium (IKMC)	www.knockoutmouse.org
The International Mouse Phenotyping Consortium (IMPC)	www.mousephenotype.org
The Knockout Mouse Project (KOMP)	www.komp.org
The Knockout Mouse Phenotyping Programme (KOMP2)	http://commonfund.nih.gov/KOMP2/
The Wellcome Trust Sanger Institute Mouse Genetics Project (SANGER-MGP)	http://www.sanger.ac.uk/mouseportal/

The European Mouse Clinics organised the EUMORPHIA programme in order to develop new mouse phenotyping approaches from 2002 to 2006. This led to the definition of the European Mouse Phenotyping Resource of Standardised Screens (EMPRESS), with standard operating procedures (SOPs) for mouse functional analysis (Brown et al. 2005). Using the knowledge gained and the acquired experience of large-scale chemical mutagenesis programmes (Hrabé de Angelis et al. 2000; Nolan et al. 2000), the European Mouse Disease Clinic (EUMODIC) brought together four European mouse clinics, the MRC in Harwell (UK), the German Mouse Clinic in Munich (Gailus-Durner et al. 2005), the Institut Clinique de la Souris (ICS) in Strasbourg-Illkirch (France), and the Wellcome Trust Sanger Institute in Hinxton, which also launched the Mouse Genetics Project (SANGER-MGP) to produce large-scale standardized mouse phenotyping data. With the EUCOMM programme commencing a year before to produce mutant ES cells in up to 8,000 mouse genes, it was a perfect opportunity to take advantage of the targeted ES cells generated by this consortium to generate mouse mutant lines and to phenotype them.

After four years of activity both programmes together produced 665 new mouse lines and the number continues to increase. The high-throughput production and phenotyping assist in deciphering the function of genes with no prior functional annotation and enhance the understanding of genes with already known functions, moving a step further in building an encyclopaedia of mammalian gene function in the mouse.

## From mutant ES cells to mouse lines

In order to carry out standardized and comprehensive phenotyping, both the EUMODIC and the SANGER-MGP programmes preferentially used ES cells from EUCOMM and KOMP programmes. These programmes started in 2006 to produce mutant ES cells with a worldwide target of 16,500 mouse genes. They used standardized procedures to generate mutant loci for genes of interest in B6 N ES cells. This created the perfect resource for the production of isogenic mutant lines in a standardized way and to carry out phenotyping.

As a consequence, 83 % (665/799) of the mouse lines were generated for the SANGER-MGP and the EUMODIC programmes from the EUCOMM and KOMP ES cell resources, respectively. The majority of mouse lines were carrying the knockout first allele named “tm1a” in which the transcription of a locus of interest is blocked by the insertion of a lacZ minigene with a strong polyA sequence just before a critical exon. A neomycin selection cassette was found after the reporter sequence. Its expression was driven either by the endogenous gene promoter (promoterless tm1a) with the β-galactosidase fused to the neomycin resistance protein with a T2A cleavage site, or by a specific promoter (promoter-driven tm1a) (Friedel et al. 2007; Skarnes et al. 2011). The tm1a allele creates a premature termination of the transcript with an interruption of the coding sequence and in most cases is associated with mRNA decay and the expression of the reporter gene. The knockout first conditional ready allele is certainly very valuable as it is possible to convert the tm1a allele to a conditional ready tm1c allele and phenotype a lethal embryonic gene by breeding the homozygous conditional mouse with a cell- or tissue-specific Cre or inducible CreERT2 line (Birling et al. 2009). Another advantage is that the lacZ sequence from the tm1a alleles is driven by the endogenous promoter allowing evaluation of cells that express the targeted gene. Furthermore, the ES cell resources were based on B6 N ES cells; this allows the generation of mouse lines in a pure genetic background as the chimeras were bred to that strain. More detailed information regarding EUCOMM and KOMP clones but also TIGM and NorCOMM mutant ES cell lines is found at the IKMC web portal (http://www.knockoutmouse.org/about) and in detail elsewhere (Ringwald et al. 2011).

The targeted genes from the ES cell resources were chosen with input from the scientific community. They were also selected because of absence of an available (conditional) mouse line in the public domain or phenotyping data. Mutant ES cells were produced, validated, and verified prior to injection at the mouse clinics. Both phenotyping programmes were keen on evaluating and improving the applicability of the mouse production and phenotyping schemes. The remaining mouse models (134/799) came from individual laboratories. They were chosen for their scientific interest, with the commitment of their owner to make the phenotyping data available as it became available. They were also pilot experiments for the phenotyping pipelines. Interestingly, different types of alleles were analysed, including null alleles, gene trap, ENU-induced mutations, large-scale deletion, and transchromosomic mice, underlying the general interest in the broad-based phenotyping effort.

The mutant mouse lines were generated from the ES cell resources using different injection strategies, using either BALB/cN or C57BL/6Brd-*Tyr* ^*c-Brd*^ blastocyst donors, with the aim of generating high rates of germline transmission (GLT). The injection parameters (e.g., number of injected cells per embryo, culture media conditions) were optimised. A minimum of 35 % and up to 70 % of GLT was obtained in the different centres. In order to simplify the breeding schemes using C57BL/6 N blastocysts, it was decided that the dominant agouti coat colour gene would be restored in the basic JM8 cells by targeted repair of the B6 nonagouti mutation (Pettitt et al. 2009). The EUCOMM and KOMP ES cell resource contains different sublines of the original ES cell line JM8 (JM8.F4, JM8.N6), with additional lines carrying the restored agouti allele (JM8A3.N1 and JM8A1.N3). Pettitt et al. (2009) presented a figure illustrating the coat colour of mice and their offspring when creating mouse lines using the different JM8 sublines. Eighty-nine percent of the mouse lines generated from the IKMC consortium ES cells and phenotyped in the EUMODIC consortium originated from the nonagouti B6 N ES cells. In total, 665 mouse lines were generated from the mutant ES cell resources. At the ICS, germline transmission was achieved, with a median time for the age of the mouse chimera of 109 days and a minimum of 65 days; 95.5 % of the GLTs were obtained by 166 days.

The tm1a allele or knockout first allele is potentially a nonexpressed (null) form in which transcription of the targeted gene is stopped. However, RT-qPCR performed on different targeted mouse models showed that in some mouse models or ES cells the polyA did not entirely stop the transcription (data not shown). In a few cases a splicing of the selection cassette was observed resulting in production of a low-level wild-type transcript (data not shown). The potential of generating a knockdown instead of a real knockout exists, even if the incidence of detecting a small amount of wild-type protein is not truly assessed. As a result of this, the IMPC consortium has decided to remove the floxed coding region prior to phenotypic analysis of future projects (especially for promoter-containing targeting cassettes as the promoter-driven neomycin is also removed) to be sure that true knockout models are being phenotyped.

## From germline transmission to cohorts

Generating the phenotyping cohort is a critical step in the process of phenotyping, from the scientific and welfare perspectives. Space and costs are bottleneck issues during the process and are affected by several components, including cohort size, age-matching animals, control strategy, genetic background, power of the tests, and statistical management of the data.

**Fig. 1:**
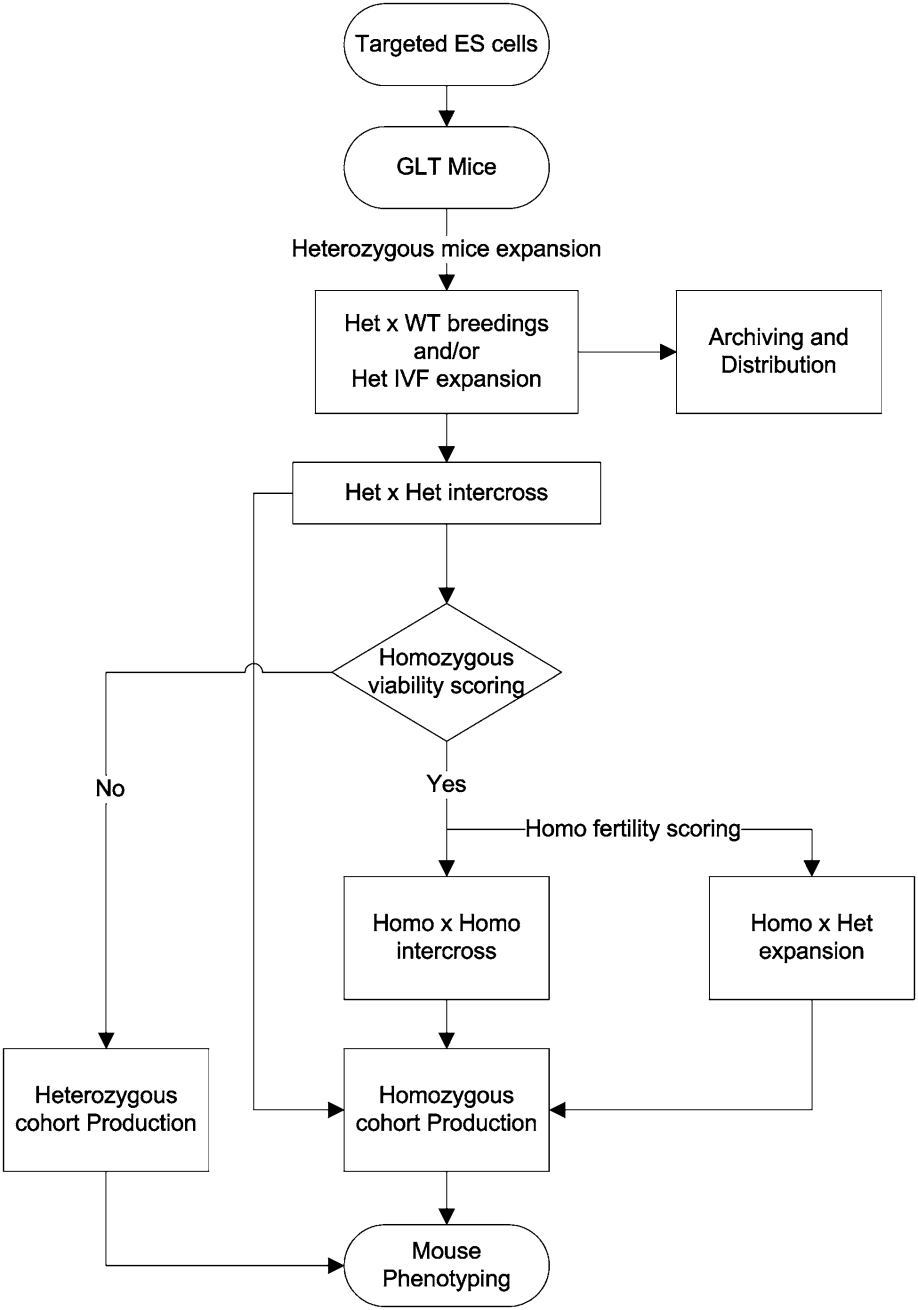
Flowchart for ES cells injection and mouse breeding strategies for cohort production. Targeted ES cells were injected to generate chimeric animals. After germline transmission of the mutant allele, the heterozygous mice were bred for pedigree expansion, archiving, distribution, and cohort production. Depending on the fertility score, heterozygous or homozygous matings were used to generate cohorts of animals for mouse phenotyping

The genetic background strongly contributes to phenotypic variations with a major impact on large biological functions in genetically engineered mice. It could affect the expression or penetrance of a given phenotype (Champy et al. 2008; Doetschman 2009; Fitch et al. 2003; Ramírez-Solis et al. 1993; Threadgill et al. 1995). To minimize this impact, both high-throughput phenotyping projects established the mutations on a pure B6 N background. The direct comparison of mutants with an appropriately matched control population of mice therefore was possible. Not only was the background uniform, but as a consequence of the breeding scheme, the controls and the mutants differed only by a limited number of breeding generations (Fig. 1). These strategies offered two advantages: first, the production of a baseline data collection to evaluate the variance, and, second, the facilitation of the interlaboratory comparison. However, one pitfall of the uniform genetic background is the potential limit on the consequences of a given mutation. Beside the ES cell resource, both programmes analysed a limited number of mouse lines coming from different laboratories with a mixed or other background strain, and in that particular case, littermates or appropriate inbred mice were used as controls.

The generation of cohorts on a large scale raises considerable logistical issues and has proven to be the major rate-limiting factor for high-throughput phenotyping. The cohort size is driven largely by the nature of the tests, and, in order to detect a significant effect (if any exists), some tests required a consistent age-matched cohort. Within EUMODIC and MGP programmes, from seven to ten mutant mice per gender had been fixed to assess the variance in phenotypes according to the power of the phenotyping tests. To produce such cohorts, the size of the breeding colonies was based on (1) this age-matched cohort requirement, (2) the reproductive strain characteristics of the parental strain used to generate the mutant strain, and (3) the various breeding schemes used between phenotyping centres.

All the mouse production and procedures were performed according to local ethical committee guidelines. Determination of animal welfare is intrinsically linked to phenotype outcome; therefore, animal welfare assessments were performed through centre-specific procedures. Nevertheless, the major bottleneck was found to be expansion of the colony, and several mouse mutant lines displayed impaired viability and fertility. Thus, embryonic or perinatal lethality and fertility were assessed as part of the overall phenotypic analysis during mouse production. Data were recorded first for the viability of homozygous offspring at weaning stage (2–4 weeks old) from heterozygous intercrosses. Viability assessment was done by genotyping a minimum of 28 offspring in order to be 95 % confident that the probability of homozygous survival is inferior or equal to 40 %. From these criteria, three outcomes were possible per mutant strain: (i) embryonic or perinatal lethality if no homozygous progeny was detected from the 28 littermates, (ii) subviability if the number of homozygotes detected was between 0 and 13 % of the 28 littermates from heterozygote intercrosses, and (iii) viability if the number of homozygotes was above 13 %. Unexpectedly, only 57 % of the 461 mouse mutant lines analysed was viable based on the results of the two programmes, with 13 % subviable and 30 % showing homozygous lethality (Table 2). The homozygous lethality was strongly dependent on the tm1a subtype, with “promoterless” targeted loci displaying 60 % of lethality compared to 28 % observed in “promoter-driven” mutant loci (χ^2^ test *P* = 2.5 × 10^−11^). In the “promoterless” targeted loci, the gene should be expressed in ES cells to drive the neomycin resistance. This correlation presumably reflects that loci expressed in ES cells have a greater probability to control critical embryonic processes and to induce lethality. This percentage of lethal genes is comparable to that observed in different chemical mutagenesis screens (Boles et al. 2009; Ching et al. 2010; Hentges et al. 2006; Kile et al. 2003; Stottmann and Beier 2010; Stottmann et al. 2011). The lethal lines and some subviable lines were further phenotyped as heterozygotes.

**Table 2:** Viability ratio in the mutant mouse lines studied during the EUMODIC and SANGER-MGP programmes

tm1a subtype	Lethal	Subviable	Viable	Total	% Lethal lines
Promoter-driven	45	22	175	242	28
Promoterless	94	37	88	219	60
Total	139	59	263	461	43
% lines	30	13	57	100	

Fertility of homozygous genetically altered mouse lines was assessed by mating at least two sexually mature mice from both genders. A mutant line was recorded as fertile using homozygous intercrosses, when offspring are generated regardless of the viability of the offspring. If fertility is uncertain in homozygous intercrosses, homozygous mutant mice are mated to nonhomozygous mice (either heterozygotes or wild types; Fig. 1). From this basic fertility assessment, mutant lines are recorded as (1) “generates offspring” if pups are produced or (2) “abnormal fertility” if no living pups are generated. Fertility assessment was also done on heterozygotes for lethal lines and labelled male, female, or both. The third outcome, not applicable (N/A), is recorded for particular classes such as imprinted genes and homozygous lethal lines. Current data indicate that overall 7 % of the mutant lines (23 of 330 tested) showed abnormal fertility.

The production of full-size cohorts was thus often the result of a compromise by finding particular homozygous mutants with impaired fertility or viability and also by centre-specific logistic constraints. As a consequence, partial-sized cohorts (on average, 3 cohorts per line) were often passed through each pipeline to reach the minimum number per gender (7 mice). Figure 1 summarizes the workflow used by the centres to optimize the mouse production for the phenotyping pipeline, which balanced the cohort size production requirement, the viability and fertility phenotype annotations, and mouse distribution. All the mutant models were archived and made available for the scientific community through the European Mouse Mutant Archive (EMMA) repository (Wilkinson et al. 2010) and KOMP repositories or directly from the SANGER-MGP.

## From mouse to phenotypes

The aim of the two phenotyping programmes was to perform a comprehensive phenotyping workflow to generate data covering most body systems, physiology, and behaviour. The EUMODIC and SANGER-MGP programmes were set up independently to carry out a comprehensive analysis and a screen of mouse mutant lines, respectively.

The EUMODIC programme used the SOPs developed within the EUMORPHIA consortium (www.eumorphia.org; Brown et al. 2005). It was shaped around two independent pipelines: the first one was devoted to morphology, metabolism, and skeletal and cardiovascular systems, while the second was oriented toward neurobehavioural and sensory systems, haematology, biochemistry, and baseline immune responses (Eumodic consortium, unpublished). The analysis began at age 9 weeks and was completed by 15 weeks of age. Its design was based on the use of two separate cohorts of mice, each composed of at least seven mutants of each gender, to detect differences in physiology or diseases, recognising that gender may have a considerable impact upon disease prevalence. It was also recommended that control mice be analysed through the phenotyping pipelines at the same time as mutants. Usually C57BL/6 N mice have been used. Mice should be born within a timeframe of 7–10 days. The phenotyping assays that have been chosen for the EMPReSSslim [European Mouse Phenotyping Resource of Standardised Screens (EMPReSS) Slim] workflow are limited, but robust, providing a relatively broad-based first pass phenotype assessment, both high-throughput and cost-effective. All SOPs are available on the EuroPhenome web site and are based on the EUMORPHIA programme (Brown et al. 2005, 2009; Morgan et al. 2010). In total, two cohorts of at least seven males and seven females were analysed through the 20 platforms of pipelines 1 and 2 (Table 3), and 413 phenotyping variables and 146 environmental or experimental metadata were recorded (Table 4).

**Table 3:** Phenotyping platforms used in the EUMODIC and WTSI-MGP programmes

Pipeline	Procedure	Age	No. of parameters	Important metadata
EUMODIC Pipeline 1	Dysmorphology	9	181	NA
	Noninvasive blood pressure	11	3	Equipment
	Calorimetry	12	10	Equipment
	Simplified IPGTT	13	3	Type of strip
	DEXA	14	10	Equipment
	X-ray	14	31	NA
	Fasted clinical chemistry	15	5	Equipment, fasting, blood collection, sample status, anaesthesia
	Heart weight	15	2	NA
EUMODIC Pipeline 2	Open field	9	18	Equipment
				Surface area
	Modified SHIRPA	9	21	NA
	Grip strength	9	7	Equipment
				Grid model
	Rotarod	10	4	Equipment
				Diameter of the rod
	Acoustic startle and PPI	11	16	Equipment
				Prepulse-pulse interval
	Hot plate	12	3	NA
	Indirect ophthalmoscopy	13	16	Topical agent(s)
	Slit lamp	13	15	NA
	Unfasted clinical chemistry	13	27	Equipment, fasting, blood collection, sample status, anaesthesia
	Haematology	13	8	
	FACS analysis	13	10	
WTSI-MGP pipeline	Open field	9	16	Equipment
				surface area
	Modified SHIRPA	9	21	NA
	Grip strength	9	6	Equipment
				Grid model
	Hot plate	10	1	Light intensity, equipment
	Dysmorphology	10	30	NA
	Indirect calorimetry	12	9	Equipment
	IPGTT	13	2	Type of strip
	DEXA	14	7	Equipment
	X-ray	14	41	NA
	Slit lamp	15	14	NA
	Ophthalmoscope	15	14	Topical agent(s)
	Haematology	16	10	Equipment, fasting, blood collection, sample status, anaesthesia
	Unfasted clinical chemistry	16	26	
	Heart dissection	16	1	NA
	FACS	16	12

**Table 4:** Lines with at least one phenotype detected in the different paradigms of the EUMODIC and WTSI-MGP programmes

EUMODIC tests and parameters	Lines with phenotypes/tested	SOP	WTSI-MGP tests and parameters	Lines with phenotypes/tested
Acoustic startle and PPI	53/313	Different	Prepulse inhibition	0/2
Body weight	59/313	Different	Weight curves	27/278
Calorimetry	57/313	Identical	Indirect calorimetry	19/278
Clinical chemistry	112/313	Identical	Plasma chemistry	54/308
Fasted clinical chemistry	57/313			
DEXA	62/313	Similar	Body composition (DEXA)	34/284
Dysmorphology	19/313	Similar	Dysmorphology	12/287
FACS analysis	56/313	Different	Peripheral blood lymphocytes	33/304
Grip strength	57/313	Similar	Grip strength	7/287
Haematology	90/313	Identical	Haematology (CBC)	27/308
Heart weight/tibia length	26/313	Identical	Heart weight	4/309
Hot plate	14/313	Identical	Hot plate	4/305
Immunoglobulin	8/313	Different	Plasma immunoglobulins	6/5
Indirect ophthalmoscopy	12/313	Different	Eye morphology (includes slit lamp and ophthalmoscopy)	12/278
Slit lamp	18/313			
Modified SHIRPA	59 / 313	Identical	Modified SHIRPA	11/287
Noninvasive blood pressure	22/313	Different	Noninvasive blood pressure	1/1
Open field	45/313	Different	Open field	122/265
Rotarod	8/313	Different		
Simplified IPGTT	20/313	Identical	Glucose tolerance (ip)	13/276
X-ray	18/313	Identical	X-ray Imaging	30/282
No phenotype detected	133/313		No phenotype detected (lines with complete data set)	159/282

The similarity of the standard operating procedure (SOP) used is indicated

**Fig. 2:**
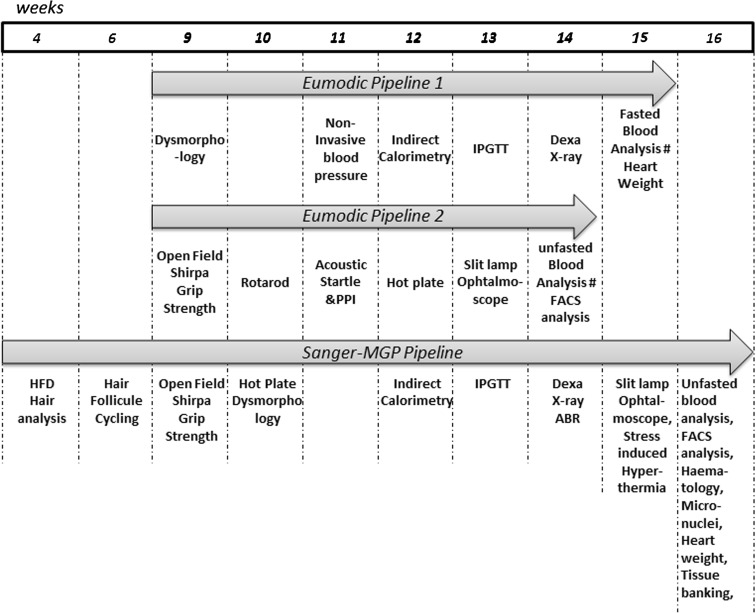
Schematic overview of the EUMODIC and MGP pipelines. The Sanger MGP pipeline started at the age of 4 weeks and ended at 16 weeks of age, whereas the EUMODIC programme encompasses two pipelines, 1 and 2, which started at 9 weeks and were completed after 7 and 6 weeks, respectively. The phenotyping platforms used in these pipelines are indicated

The SANGER-MGP pipeline was designed as a single pipeline starting at 5 weeks of age with a high-fat diet challenge, and followed by a battery of tests from week 4 to 16, as shown in Fig. 2 and Table 3. The pipeline was designed to identify a variety of models from obesity to dysmorphology with a series of 13 wide-ranging platform tests, and record a number of variables for phenotypic assessment as well as additional metadata impacting conditions or equipment. Groups of mutant animals (up to 7 males and 7 females per line) went through as they were produced, along with one control cohort each week for each genetic background. The pipeline was further enriched by additional procedures devoted to specific functions such as a challenge for immune or auditory brainstem response measurements that could be analysed during the pipeline or with a new batch of mutant cohorts (Table 5).

**Table 5:** Additional phenotypes detected with the specific tests integrated in the WTSI-MGP pipeline

WTSI-MGP specific tests	Lines with phenotypes/tested
Auditory brainstem response	15/323
General observations	18/18
MicroCT and quantitative Faxitron	12/51
Citrobacter challenge	23/229
Salmonella challenge	10/238
Micronuclei	8/197
Stress-induced hyperthermia	3/282
Tail epidermis whole mount	4/40
Eye histopathology	3/80
Skin histopathology	4/98
Brain histopathology	9/111

The two phenotyping programmes focused on basic physiological aspects, with platforms operating with nearly identical or similar procedures for 11 and slightly different ones for 8 (Table 3) with a set of common variables and metadata. Statistical analyses have been set up based on the way cohorts and controls were produced to ensure the use of appropriate tests to detect the phenodeviants when they exist. Two main approaches were applied: (1) classical inferential statistics based on the use of large baseline control sets, and (2) for the partial phenotyping group, the reference range approach was preferred, using the baseline data collection which ensured a simple and robust strategy. The reference range approach was preferentially used by the SANGER-MGP approach irrespective of the cohort size. The analysis of data also helped to refine the tests and to reduce the number of animals used in the cohorts (Karp et al. 2010).

Even though the precise timing and the local organization of the workflow varied, and differences between platforms exist between the two phenotyping programmes and the four centres involved, the efficiency of the platforms in detecting phenotype is quite similar (Eumodic consortium, unpublished). Both phenotyping pipelines were successful in identifying phenotypes in mutants for genes for which there was phenotyping annotation for at least one allele already available. Sixteen of 30 mutants analysed in the EUMODIC programme displayed phenotyping overlaps to annotations found in the Mouse Genome Informatics database (http://www.informatics.jax.org/) (Eumodic consortium, unpublished).

Taking into account the fertility and viability annotations, both programmes revealed at least one phenotype in about 83.8 % (EuroPhenome) and 76.5 % (SANGER-MGP) of the mutant lines, demonstrating the power of comprehensive phenotyping in defining functions for any gene of interest. A few additional platform screens were stopped or abandoned in the SANGER-MGP programme and replaced with new tests (Table 5). With this new panel of tests, the SANGER-MGP programme increased the coverage in phenotyping particular aspects not detailed within the common shared platforms. Where homozygote mutants were not viable, both programmes have analysed heterozygotes. Accordingly, the EUMODIC programme had a phenotypic hit rate of 71.7 % in this class, underlining the potential for the annotation of genes through heterozygote analysis.

## Future perspectives

In conclusion, the main objective of the two efforts was achieved with the generation of nearly 800 mouse models, which are available and accompanied by a broad-based phenotype analysis. Furthermore, both programmes reach similar conclusions on mutant lethality, finding that around 30 % of mouse mutants are not viable. Both programmes highlighted the efficiency of the broad-based phenotyping approaches, identifying at least one phenotype in around 80 % of the mouse mutants. The mouse clinics have provided a new set of data on gene function to the scientific community while improving the throughput and reducing the cost of the analysis. The results summarized here provide many logistical, operational, and scientific lessons that will be vital as we begin the next step in undertaking a global project of exploration of the function of genes and generation of an encyclopaedia of the mammalian genome by the IMPC.

Nevertheless, additional resources are required, most importantly the completion of the project of generating mutant ES cells for all loci in the mouse genomes: not all the genes are available yet in the IKMC. New initiatives have already begun with three new programmes—The EUCOMM-Tools for Functional Annotation of the Mouse Genome (EUCOMMTools), the Knockout Mouse Phenotyping Programme (KOMP2), and Norcommt2ls—to generate a final series of mutant ES cells, along with a new set of genetic tools for conditional genetics. Since 2011, miR knockout (KO) ES cells are also available on the IKMC web site. Prosser et al. (2011) have developed a resource of vectors and ES cells for targeted deletion of microRNAs in mice and show how to generate, using Recombinase Mediated Cassette Exchange (RMCE) from this miR KO allele, a reporter of the miR promoter activity, the conditional KO, and how to mutate one or other miR organised within the miR clusters. Further resources will be needed for long noncoding RNAs, for point mutations, and for copy number variants, and more generally to decipher the regulatory mechanism of the transcriptional and genome organisation. In addition, it will be important to consider routes to explore heterozygous lethal or haploinsufficient mutations that escape current analyses.

In the two programmes discussed, the mutants generated so far and analysed were almost all derived from one allele, the tm1a allele. A limitation of this approach is the efficient transcription stop due to the presence of the polyA. We know from a series of experiments in the different clinics that some tm1a alleles do not lead to full expected inactivation of the targeted gene. One way to circumvent the problem is to use the tm1b allele carrying the deletion of the critical exon(s) without the removal of the lacZ sequence (or the lacZ and neo sequences in the promoterless mutants). To obtain such a tm1b allele, different universal deleters are available, but some deleters are likely to be preferred. For example, the Gt(ROSA)26Sor^tm1(ACTB-cre,-EGFP)Ics^ deleter eliminates one generation of breeding due to the maternal accumulation of the Cre in the oocytes allowing the accumulation of the recombinase during oogenesis and the direct recovery of individuals carrying the deletion without the Cre transgene (Birling et al. 2011). In addition, the line was developed on a pure C57BL/6 N genetic background and the presence of the transgene can be genotyped by GFP fluorescence.

The main outcomes of the first set of mutants that were analysed are the high level of homozygote lethality observed with over 30 % of the lines studied and the high level of phenotype annotations associated with heterozygous mutants. Nevertheless, a number of additional approaches should be considered. First, the lacZ pattern of the mutant allele should be explored. The use of tm1a or tm1b allele’s lacZ reporter enables us to study the expression of the gene with good sensitivity in heterozygotes. Of course, an inconvenience of this strategy is that regulatory elements or other genomic elements (e.g., ncRNA, miRNA) in the intron of the gene are modified as well. In addition, the locus might code for various isoforms with different promoters. If the isoform expressed in the organ of interest is different from the isoform targeted and if it uses a different (endogenous) promoter, the lacZ will not be expressed as expected. Second, the phenotyping pipeline should also integrate an embryonic screen to determine key steps of embryonic development. Some centres have already developed an embryonic lethal screening pipeline on a limited number of lines with some success.

The EUMODIC and SANGER-MGP programmes pioneered the high-throughput production and comprehensive analysis of mice knockouts. Considerable experience has been gained in the logistics and operation of phenotyping pipelines that will underpin future developments in international phenotyping programmes. For example, much information has been gathered on the effectiveness and sensitivity of phenotyping platforms that will prove valuable in the design of future pipelines, including the introduction of new assays to cover physiological systems. Along with the new partners of the IMPC, a new adult pipeline has been developed and the establishment of a unique and high-throughput embryonic pipeline is under discussion. As part of this process, additional assays for immune systems, inflammation, respiration, cognition, and behaviour have all been considered. In addition, building the research infrastructure capacities required in Europe for these large-scale efforts, as well as for access of individual researchers to high-throughput phenotyping, is the task of the pan-European Infrafrontier programme, which has been prioritized by ESFRI (European Strategy Forum on Research Infrastructures). Infrafrontier also secures sustainable access to mouse models archived in EMMA. Similar efforts are underway in North America and in Asia. With these new developments, we can expect that the upcoming large-scale phenotyping pipeline operated by the IMPC will prove even more effective in delivering broad-based phenotype information on a wide variety of body systems and physiological functions.
